# Early Poststroke Rehabilitation Using a Robotic Tilt-Table Stepper and Functional Electrical Stimulation

**DOI:** 10.1155/2013/946056

**Published:** 2013-04-14

**Authors:** Alexey N. Kuznetsov, Natalia V. Rybalko, Vadim D. Daminov, Andreas R. Luft

**Affiliations:** ^1^Department of Neurology and Neurosurgery, National Pirogov Centre of Therapy and Surgery, Nignaya Pervomaiskaya Street 70, Moscow 105203, Russia; ^2^Clinical Neurorehabilitation, Department of Neurology, University of Zurich, 8091 Zurich, Switzerland

## Abstract

*Background*. Stroke frequently leaves survivors with hemiparesis. To prevent persistent deficits, rehabilitation may be more effective if started early. Early training is often limited because of orthostatic reactions. Tilt-table stepping robots and functional electrical stimulation (FES) may prevent these reactions. *Objective*. This controlled convenience sample study compares safety and feasibility of robotic tilt-table training plus FES (ROBO-FES) and robotic tilt-table training (ROBO) against tilt-table training alone (control). A preliminary assessment of efficacy is performed. *Methods*. Hemiparetic ischemic stroke survivors (age 58.3 ± 1.2 years, 4.6 ± 1.2 days after stroke) were assigned to 30 days of ROBO-FES (*n* = 38), ROBO (*n* = 35), or control (*n* = 31) in addition to conventional physical therapy. Impedance cardiography and transcranial doppler sonography were performed before, during, and after training. Hemiparesis was assessed using the British Medical Research Council (MRC) strength scale. *Results*. No serious adverse events occurred; 8 patients in the tilt-table group prematurely quit the study because of orthostatic reactions. Blood pressure and CBFV dipped <10% during robot training. In 52% of controls mean arterial pressure decreased by ≥20%. ROBO-FES increased leg strength by 1.97 ± 0.88 points, ROBO by 1.50 ± 0.85 more than control (1.03 ± 0.61, *P* < 0.05). CBFV increased in both robotic groups more than in controls (*P* < 0.05). *Conclusions*. Robotic tilt-table exercise with or without FES is safe and may be more effective in improving leg strength and cerebral blood flow than tilt table alone.

## 1. Introduction

Stroke poses enormous medical and social problems to societies worldwide. Stroke incidence ranges between 101 and 285 per 100.000 inhabitants per year depending on continent and region [1]. Ninety percent of stroke survivors are left with deficits, and about one-third remain dependent in activities of daily living [2]. Starting rehabilitation as soon as possible, therefore, is a key goal of stroke care. Poor outcome is more likely if rehabilitation is delayed [3]. Early mobilization is an important step to further recovery. The AVERT trial in which subjects was mobilized out of bed within 24 hours after a stroke suggests that early mobilization is safe [4] and effective in reducing long-term dependency [5]. Still, in most stroke services patients stay in bed for prolonged periods of time because of circulatory instability or limited patient cooperation.

Robots can help early mobilization. Robots can assist the patient with altered states of vigilance enabling movement repetitions that may induce central nervous system reorganization processes that lead to functional recovery [6]. Robotic tilt tables may bring the patient in an vertical position while moving their legs to prevent blood pressure drops. Robotic therapy has been safely and successfully used in acute stroke patients in the past [7]. Functional electrical stimulation (FES) is an alternative strategy to assist movements in patient that cannot move actively. Functional electrical stimulation (FES) induces muscle contractions via electrodes placed on the skin [8] or implanted over the target muscles [9]. FES has been successfully used to assist walking [10] and reaching movements [11].

Neither for robot nor FES therapy, safety, feasibility, and efficacy have been demonstrated in patients during the acute phase after stroke. The objective here was to assess safety—especially with respect to orthostatic reactions—of robot-assisted verticalization with and without FES shortly after a stroke. A preliminary assessment of efficacy was also conducted. 

## 2. Methods

### 2.1. Participants

This study evaluates safety and feasibility of robotic/FES acute rehabilitation in a convenience sample of patients that were assigned to one of three groups allowing for between-group (control) comparisons. The trial does not fulfill the CONSORT criteria of a randomized controlled trial and was therefore not registered.

Inclusion criteria were ischemic stroke in the territory of the middle cerebral artery not longer than 7 days before enrollment participated in the trial. Patients with hemiparesis 4 or less points on the MRC scale were included. The diagnosis of ischemic stroke was verified by CT or MRI.

We excluded patients with an NIHSS > 16 points, because we assumed that the severity of the disease would interfere with the accessibility of the patient for training. Additional exclusion criteria were severe cognitive impairment (MMSE < 10), severe aphasia, blood pressure (BP) persistently higher then 160/100 mmHg or lower than 90/60 mmHg, internal carotid artery stenosis > 60% by NASCET criteria, severe cardiac disease, intracardiac thrombus, severe medical conditions, contractures of lower extremities, thrombophlebitis, and lower extremity deep vein thrombosis. All patients gave written informed consent. Experimental protocols were approved by the institutional ethics committee.

All patients received acute care for ischemic stroke according to institutional protocols in accordance with international guidelines. Conventional rehabilitation consisting of physical and physiotherapy (physical exercises, massage, and mechanotherapy) was delivered for 30 days (0.5–1 h/work day) other in addition to experimental and control treatments.

In parallel to conventional therapy, participants received either robotic tilt table (Erigo, Hocoma AG, Volketswil, Switzerland) combined with FES (ROBO-FES), robotic tilt-table alone (ROBO), or tilt-table training alone (control). Group assignment was determined by day of admission; that is, patients admitted on Mondays or Thursdays were assigned to ROBO-FES, those admitted on Tuesdays and Fridays to ROBO, and those admitted on Wednesdays and Saturdays to control.

### 2.2. Apparatus

**Figure 1:**
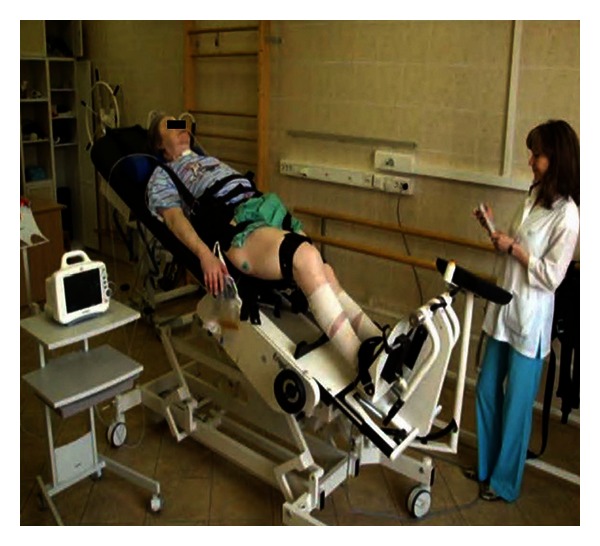
The device combining tilt-table functionality with stepping movement is shown.

The robotic tilt table (Erigo, Hocoma AG, Volketswil, Switzerland, Figure 1) consists of a stretcher that can be tilted between 0°–80° and foot plates with integrated springs for leg loading. Foot plates perform stepping-like movements. Training on the Erigo combines mobilization out of bed, body verticalization, and rhythmic leg movement with cyclic loading.

### 2.3. Training Protocols

Robotic therapy was administered according to the following protocol. Patients received from 20 to 30 minutes of training daily over a period of 30 days. During first three training sessions patients were gradually verticalized from 10 to 30 degrees, and stepping was performed at a rate of 38–40 steps per minute. Loading of the legs was either passive or passive-active. By session 5, verticalization was then increased to 60 degrees and stepping to 40–56 steps per minute. By session 24, verticalization was further increased to 80 degrees as tolerated by the patient. The control group was moved in vertical position on the tilt table using an identical protocol except that no stepping or FES was performed.

The ROBO-FES group received additional FES using a 6 channel stimulator (Motionstim 8, Medel GmbH, Hamburg, Germany). Electrodes were placed over biceps femoris, quadriceps femoris and gastrocnemius of either leg. The stimulation was synchronized with robotic leg movements: biceps femoris, and gastrocnemius muscles were stimulated at the time of leg flexion; quadriceps femoris was simulated at the time of leg extension. Strength of stimulation varied between 5 and 100 mA. 

### 2.4. Outcome Measures

Hemiparesis was measured using British MRC strength scale. Systolic and diastolic blood pressure and stroke volume were measured using impedance cardiography (Cardioscreen 1000, Niccomo PC, USA). Cerebral blood flow was assessed using transcranial doppler ultrasonography (TCD) of the middle cerebral artery in the hemisphere affected by the stroke. TCD was performed using pulse-wave doppler and a range-gated 2 MHz probe (Nicolet, CareFusion, Rolle, Switzerland) that was fixated over the temporal bone window by a helmet (Spencer Technologies, Seattle, WA, USA). The MCA was insonated at a depth of 55 mm. Maximum systolic velocity, minimum diastolic velocity, and the indexes of peripheral resistance (pulsatility index (PI) and resistance index (RI)) were measured. Both impedance cardiography and TCD were performed over a period of 30 minutes. All outcome measures were collected within 1-2 days before starting and after ending the 30-day training period. Additionally, cerebral blood flow and hemodynamic parameters were measured during the first training session.

### 2.5. Statistical Analyses

SPSS (version 11, SPSS Inc, Chicago, IL, USA) was used for all statistical analyses. Because of the small sample size in each group, nonparametric tests (Kruskal-Wallis test, KW) were used to assess the effect of group on the change of each outcome variable (postintervention baseline). Because no primary outcome variable was prespecified we present the results also corrected for multiple comparisons using Benferroni's correction for *n* = 9 tests (strength, Barthel, systolic and diastolic MCA velocity, PI, RI, and systolic and diastolic blood pressure). Dunn's multiple comparison test was used for comparisons between each intervention group and the control group. In case of significant between-group differences at baseline, the baseline variable was included as a covariate in the model. Two-tailed *P* values < 0.05 were considered significant.

## 3. Results

**Figure 2:**
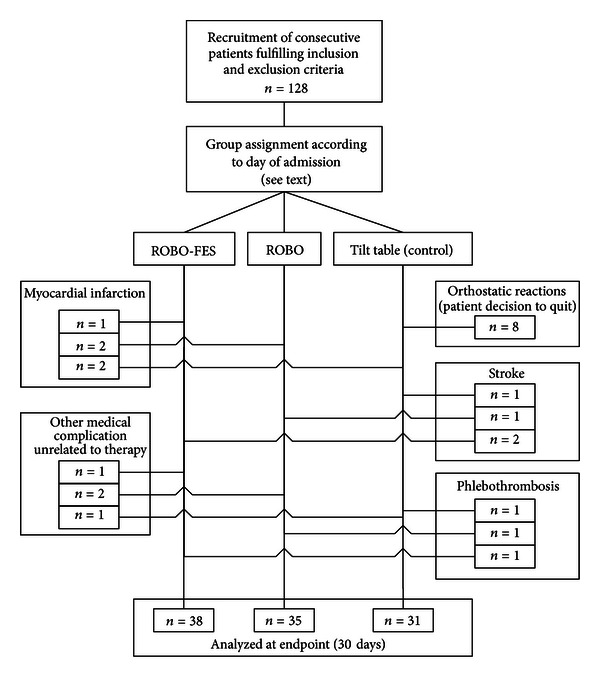
Participant flow through the study protocol.

A total of 128 patients were recruited. 24 patients dropped out for different reasons (see Figure 2). Adverse events with a relationship to therapy, that is, orthostatic reactions leading to dizziness, occurred in the tilt-table group only (*n* = 8). These eight patients chose to terminate the study and were excluded from further analyses. 

Of the 104 patients who completed the study, 38 were treated with ROBO-FES, 35 with ROBO, and 31 with tilt table (controls). Mean age of the 104 subjects was 58.3 ± 1.2 years (mean ± SD). In 52 patients, stroke affected the right hemisphere. Patient demographics, cardiovascular risk parameters, and baseline impairment and dependency pre group are presented in Table 1. In addition to hemiparesis, 34% of patients had sensory deficits, and 25% suffered from ataxia. Training was started on average 4.6 ± 1.2 days after the stroke.

### 3.1. Effects of 30 Days of Training

**Figure 3:**
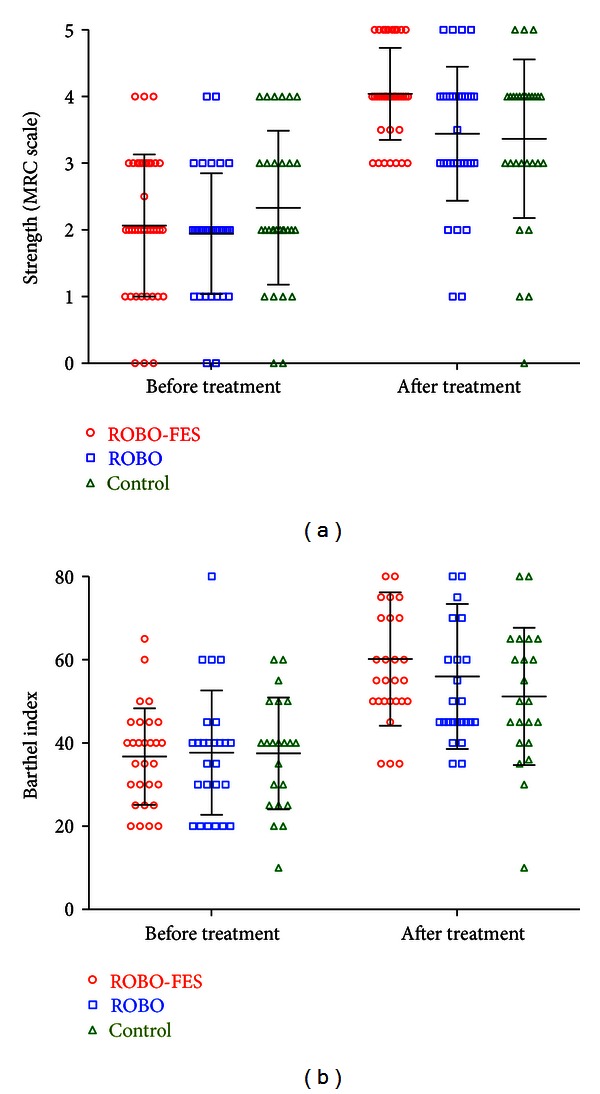
Changes in leg strength measured by the MRC scale (a) and Barthel index (b) over the course of treatment. Strength gains were significantly higher in ROBO-FES and ROBO as compared to control. Effects of group on the Barthel index did not reach statistical significance. Error bars indicate standard deviations.

Training induced changes in outcome measures are summarized in Table 2. Leg strength improved differently between treatment protocols (KW statistic 19.3, *P* < 0.0001, Bonferroni corrected *P* < 0.0001, Figure 3(a)). In ROBO-FES leg strength increased more than in control (Dunn's test *P* < 0.05). The difference between ROBO and control and between both robotic groups was not significant. Similarly, the Barthel index increased in ROBO-FES more than in ROBO or control, but the overall effect of group was not significant (KW statistic 5.92, *P* = 0.0518, Bonferroni-corrected *P* = 0.46, Figure 3(b)).

**Figure 4:**
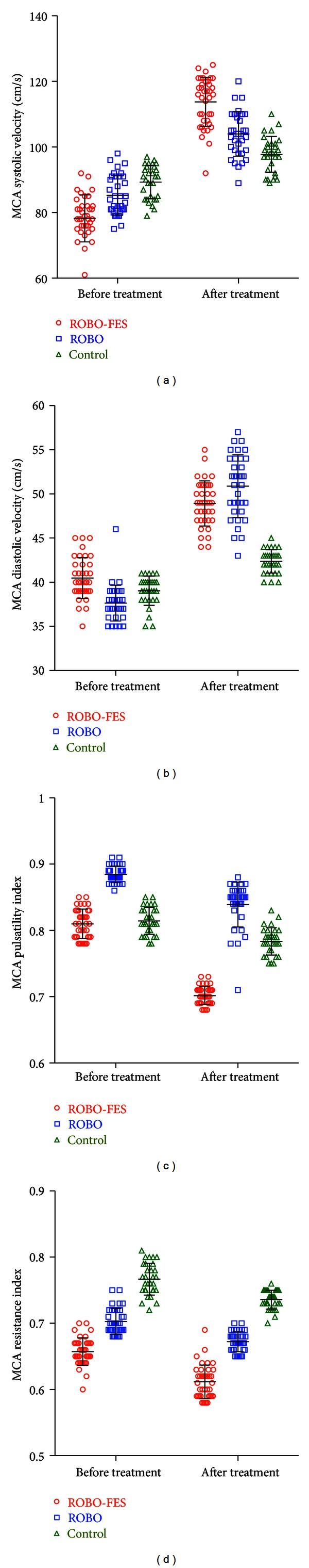
Changes in cerebral blood flow parameters over the course of treatment. (a) and (b) Systolic and diastolic blood flow velocity in the middle cerebral artery (MCA) of the affected hemisphere increased in robotic groups more than in control. (c) and (d) Indices of pulsatility and vascular resistance were mainly improved by ROBO-FES, while ROBO training had similar effects as compared with control. Error bars indicate standard deviations.

The groups showed different changes in MCA blood flow velocity over the 30-day training period (for systolic, KW 69.2, and diastolic velocity, KW 67.4, both *P* < 0.0001, Bonferroni-corrected *P* = 0.0002, Figures 4(a) and 3(b)). Cerebral blood flow velocity changed most after ROBO-FES followed by ROBO and control (all differences between groups were significant for systolic and diastolic velocity). The pulsatility index (PI) improved in ROBO-FES more than in control and ROBO (group effect KW 60.1, *P* < 0.0001, Bonferroni-corrected *P* < 0.0001, Dunn's post hoc test *P* < 0.05; difference between ROBO and control was not significant). There was a significant group effect on the resistance index (RI, KW 6.14, *P* = 0.0465), but the result was no longer significant when multiple comparison correction was applied (*P* = 0.42). Dunn's post hoc tests for comparisons between groups were insignificant (Figures 4(c) and 3(d)). All models on MCA blood flow parameters gave similar results if the respective baseline variable was included as a covariate into the model.

**Figure 5:**
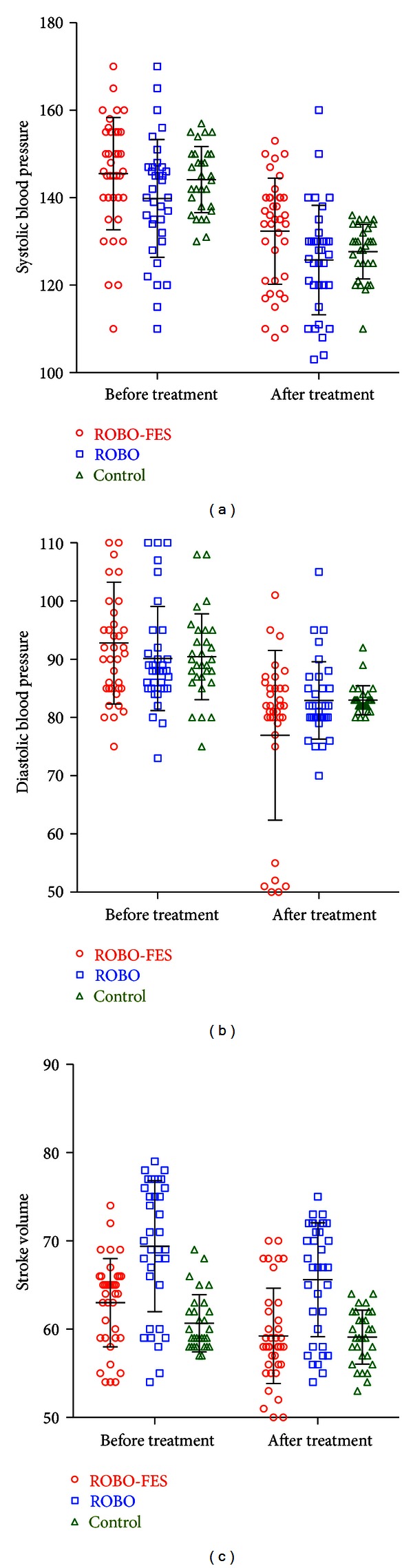
Changes in systemic blood pressure over the course of training. Systemic blood pressure (a, b) and stroke volume (c) similarly decreased in all group. Only diastolic BP was more affected by ROBO-FES than by other interventions. Error bars indicate standard deviations.

Diastolic but not systolic blood pressure was affected by group (KW 7.76, *P* = 0.0009, Bonferroni-corrected *P* = 0.0081, baseline diastolic pressure included as covariate, post hoc difference between ROBO-FES and ROBO, *P* < 0.05, Figures 5(a) and 4(b). Similarly, group effects on stroke volume were only significant (KW 6.97, *P* = 0.0306) if multiple comparisons were not corrected for (*P* = 0.28). There was a significant difference between groups at baseline (*P* < 0.0001). If baseline values were entered as a covariate into an ANOVA model, the group effect disappeared (*P* = 0.075, uncorrected, Figure 5(c)).

### 3.2. Cerebral Blood Flow and Blood Pressure during Training

**Figure 6:**
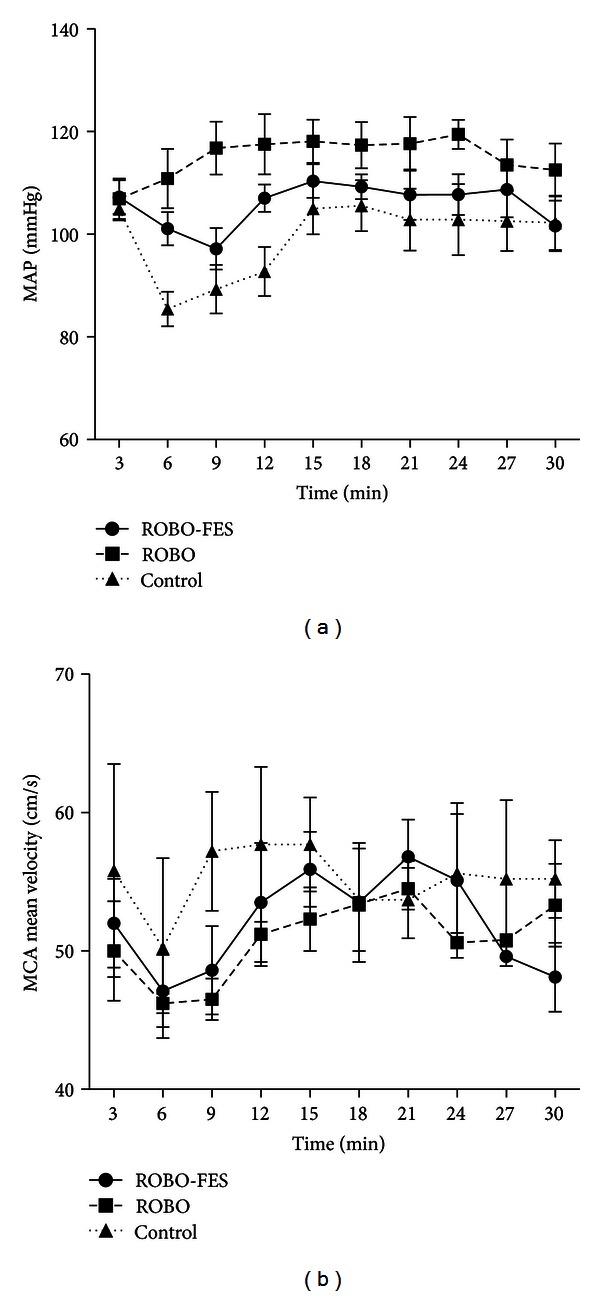
Intrasession evolution of mean arterial pressure (MAP) and mean cerebral blood flow velocity (CBFV) in the MCA of the affected hemisphere during training session 1 (values are averaged in 3 min-time windows). (a) MAP shows an initial dip in control and ROBO-FES groups during the first 9 min of training and returned to baseline thereafter. (b) Similarly, mean CBFV only decreased in the first 6–9 min and then quickly returned to baseline. Error bars indicate SD.

Mean arterial pressure dropped at the beginning of session 1 by 5.7%, 0%, and 28% in ROBO-FES, ROBO, and control groups, respectively. MAP quickly returned to baseline thereafter (Figure 6(a)). None of the patients in the ROBO and ROBO-FES groups had postural hypotension or orthostatic reactions when put in a vertical position as defined by a decrease in systolic blood pressure of ≥20 mmHg and in diastolic pressure of ≥10 mmHg [12]. But 52% of control subjects showed hypotension to a minimum of 105/76 mmHg. In none of the patients, this pressure drop was considered significant enough to mandate termination of training. Nevertheless eight patients in the control group decided to discontinue and dropped out of the study (these are not included in the analysis). Blood pressure changes occurred only during the first 9 minutes of verticalization.

Mean MCA blood flow velocity dipped by 9.4%, 7.6%, and 10% during the first 9 min of training in ROBO-FES, ROBO, and control groups, respectively. Subsequently, blood flow remained stable throughout the training session (Figure 6(b)).

## 4. Discussion

This study suggests that robotic tilt-table training (ROBO) in combination with functional electrical stimulation (FES) is safe and may improve strength as well as cerebral blood flow and blood pressure. As an addition to conventional physiotherapy, ROBO-FES was more effective than verticalization only using a tilt table. ROBO training without FES induced smaller changes than ROBO-FES but was still superior to tilt-table in improving leg strength and cerebral blood flow.

Early mobilization out of bed is generally desired to prevent systemic complications after stroke [13]. The AVERT randomized controlled trial suggests that very early mobilization within 24 hours after stroke benefits mobility and functional independence one year later [5]. Mobilization is sometimes difficult to achieve: reduced vigilance and cooperability, trunk instability, pusher symptoms, orthostatic hypotension, logistic, or spatial constraints can delay mobilization. Simple devices to facilitate body verticalization are tilt tables, but their value is limited because they do not encourage limb movement and may provoke hypotension. Robotic tilt tables like the one used here, move the legs passively in a pattern that resembles stepping and simulate body weight on leg joints by pushing against the legs. Functional electrical stimulation can activate leg muscles to generate stepping-like patterns. In combination, these interventions simulate the signature of sensory feedback of upright walking.

Different rationales have been proposed for the use of robotic mobilization and FES. One is to improve orthostatic hypotension, which is common in patients with spinal cord or traumatic brain injury. Venous return and, hence, cardiac output increase during passive leg cycle exercise in healthy and in subjects with spinal cord injuries [14]. Similar effects are observed with functional electrical stimulation to induce leg cycling [15]. Case series indeed suggest that robotic verticalization is associated with fewer hypotensive episodes than tilt-table training [16]. Our study strongly supports these findings: no episodes of marked hypotension were observed during verticalization on a robotic tilt table with or without FES. In contrast, more than half of the patients in the tilt-table group showed decreases in blood pressure of 20% or more. Therefore, the addition of stepping movements may prevent orthostatic responses to early verticalization. This finding is possibly caused by a better venous return through stepping movements.

In addition to orthostatic reactions, verticalization may cause harm by decreasing cerebral blood flow, which depends on head position. Lowering the head has been suggested to improve blood flow in the acute phase after stroke [17]. We therefore measured cerebral blood flow velocity during training. A <10% drop in cerebral blood flow occurred at the beginning of the session in all groups. Despite this drop, patients remained asymptomatic.

A second rationale is to improve vigilance. In comatose or vegetative patients, verticalization may improve the regaining of consciousness (reviewed in [18]). Whether the addition of stepping or FES to tilt-table exposure increases this effect, it is unknown. None of our patients was comatose; therefore, we cannot speak to this rationale.

A third rationale is to reduce motor impairment by preventing long-term spasticity and improving strength. Our findings suggest that leg strength improves more after ROBO-FES as compared with ROBO and control. However, the study was not designed to evaluate the efficacy of the therapies to improve leg paresis. Its limitations (see the following) limit the interpretability of these findings.

Thirty days of training in ROBO and ROBO-FES groups did induce lasting changes in cerebral perfusion. Comparing cerebral blood flow to the lesioned hemisphere before and after training showed greater improvement in the robotic groups. Pulsatility and vascular resistance improved likewise. Improved perfusion in certain brain regions has been observed in chronic stroke patients after constraint induced movement therapy using single photon emission computed tomography. These findings have been interpreted as evidence of therapy-related cortical reorganization [19]. This interpretation unlikely holds here, because MCA blood flow velocity as measured by TCD is insensitive to small changes in regional perfusion. More likely our findings indicate hyperperfusion or luxury perfusion in robotic groups. While hyperperfusion may be detrimental in the acute phase (so-called reperfusion injury, reviewed in [20]), luxury perfusion that occurs in up to 83% of patients by week 3 after stroke [10] indicates positive outcomes ([10], reviewed in [20]).

In contrast to cerebral blood flow velocity, systemic blood pressure declined in all three groups over the 30-day training period, reflecting the expected time course of blood pressure evolution after acute stroke [21]. We cannot explain why diastolic blood pressure decreased in ROBO-FES more than in other groups. We assume a coincidental finding, considering the fact that systolic pressure showed comparable changes between groups.

This study has several limitations. Most importantly, a relatively crude assessment of strength using the MRC scale was the only functional outcome. As our main objective was to address safety, we focused on adverse events and physiological parameters. A simple functional outcome was added because we expected logistic problems with collecting a more sophisticated assessment of impairment, such as the Fugl-Meyer scale. Another limitation is the lack of followup data. Both questions—functional outcome and maintenance of therapy effects—are important to answer in future studies. A third limitation is the lack of randomization. This may explain the differences between the groups observed at baseline for various outcome measures. However, because we could not identify a systematic difference in the assignment to group, the differences may also have occurred by chance.

## 5. Conclusions

In conclusion, this controlled study suggests that robotic tilt-table training with and without functional electrical stimulation is safe and feasible in the postacute period after stroke. It provides preliminary evidence that in combination with conventional physical therapy this training is more effective in improving leg strength as compared with tilt-table training alone.

## Figures and Tables

**Table 1:** Patient demographics and cardiovascular risk variables.

	ROBO-FES	ROBO	Control	P*
Number	39	35	30	
Age (mean, SD)	61.0, 9.33	59.0, 8.09	57.0, 8.26	0.86
Gender (number of females)	18	16	14	1.0
NIHSS at enrollment	13.4	12.4	13.0	0.88
Hypertension (%)	30	26	28	0.86
Cardiac arrhythmia (%)	17	8	19	0.38
DM II (%)	7	8	6	0.96
Smoking (%)	20	18	15	0.88
Obesity (BMI > 30) (%)	15	20	19	0.84

*P of between-group differences in the means of the variables.

**Table 2:** Change in outcome measure within each group.

	ROBO-FES		ROBO		Control		Between group *P*	Mult comp corrected *P*
	Baseline	Post	Baseline	Post	Baseline	Post
Strength	2.10	4.00	1.90	3.40	2.30	3.40	<0.0001	<0.0001
Absolute Δ ± SD	1.97	0.88	1.50	1.50	1.03	0.61		
Barthel index	36.7	60.2	37.7	56.0	37.5	51.2	0.0518	ns
Absolute Δ ± SD	23.4	15.9	18.3	12.8	13.7	9.62		
MCA systolic CBFV (cm/sec)	78.3	114	85.3	104	89.3	97.8	<0.0001	0.0002
Absolute Δ ± SD	35.5	8.04	18.7	8.97	8.5	5.92		
MCA dioastolic CBFV (cm/sec)	40.5	48.9	37.6	50.9	39.0	42.4	<0.0001	0.0002
Absolute Δ ± SD	8.45	3.11	13.3	3.42	3.3	1.94		
PI	0.81	0.70	0.88	0.84	0.81	0.78	<0.0001	<0.0001
Absolute Δ ± SD	−0.11	0.03	−0.05	0.03	−0.03	0.02		
RI	0.66	0.61	0.70	0.67	0.77	0.74	0.0456	ns
Absolute Δ ± SD	−0.05	0.03	−0.03	0.03	−0.03	0.02		
Systolic BP (mmHg)	146	132	140	126	144	128	0.24	ns
Absolute Δ ± SD	−13.1	10.6	−14.1	13.9	−16.5	7.54		
Diastolic BP (mmHg)	92.8	76.9	90.1	82.9	90.4	83.0	0.0009	0.0081
Absolute Δ ± SD	−15.8	14.4	−7.17	7.05	−7.47	5.84		
Stroke volume (mL)	63.0	59.2	69.4	65.6	60.7	59.1	0.075	ns
Absolute Δ ± SD	−3.76	4.69	−3.80	3.91	−1.57	2.85		
